# Post-Acute Dyslipidemia and Abnormal Body Mass Index in Children and Adolescents with COVID-19: A Cohort Study from the RECOVER Initiative

**DOI:** 10.1016/j.jpeds.2026.114996

**Published:** 2026-01-19

**Authors:** Yuqing Lei, Ting Zhou, Bingyu Zhang, Dazheng Zhang, Huilin Tang, Jiajie Chen, Qiong Wu, Lu Li, L. Charles Bailey, Michael J. Becich, Saul Blecker, Dimitri A. Christakis, Daniel Fort, Sharon J. Herring, Wenke Hwang, Amrik Singh Khalsa, Susan Kim, David M. Liebovitz, Abu Saleh Mohammad Mosa, Suchitra Rao, Soumitra Sengupta, Xing Song, Yacob G. Tedla, Ravi Jhaveri, Caren Mangarelli, Christopher B. Forrest, Yong Chen

**Affiliations:** 1 The Center for Health AI and Synthesis of Evidence (CHASE), University of Pennsylvania, Philadelphia, PA;; 2 Department of Biostatistics, Epidemiology, and Informatics, University of Pennsylvania Perelman School of Medicine, Philadelphia, PA;; 3 The Graduate Group in Applied Mathematics and Computational Science, School of Arts and Sciences, University of Pennsylvania, Philadelphia, PA;; 4 Department of Biostatistics and Health Data Science, University of Pittsburgh, Pittsburgh, PA;; 5 Applied Clinical Research Center, Children’s Hospital of Philadelphia, Philadelphia, PA;; 6 Department of Pediatrics, Children’s Hospital of Philadelphia, Perelman School of Medicine, University of Pennsylvania, Philadelphia;; 7 Department of Biomedical Informatics, University of Pittsburgh School of Medicine, Pittsburgh, PA;; 8 Department of Population Health, NYU Grossman School of Medicine, New York, NY;; 9 Center for Child Health, Behavior and Development, Seattle Children’s Research Institute, Seattle, WA;; 10 Center for Outcomes Research, Ochsner Health, New Orleans, LA;; 11 Department of Population Health and Urban Bioethics, Program for Maternal Health Equity, Center for Urban Bioethics, Center for Obesity Research and Education, College of Public Health, Lewis Katz School of Medicine at Temple University, Philadelphia, PA;; 12 Department of Public Health Sciences, Penn State University College of Medicine, Hershey, PA;; 13 Division of Primary Care Pediatrics, Center for Child Health Equity and Outcomes Research, Abigail Wexner Research Institute, Nationwide Children’s Hospital, Columbus, OH;; 14 Department of Pediatrics, College of Medicine, The Ohio State University, Columbus, OH;; 15 Division of Rheumatology, University of California, San Francisco, Benioff Children’s Hospital, San Francisco, CA;; 16 Department of Medicine, Northwestern University Feinberg School of Medicine, Chicago, IL;; 17 Clinical Research Informatics, Heersink School of Medicine, University of Alabama at Birmingham, Birmingham, AL;; 18 Department of Pediatrics, University of Colorado School of Medicine and Children’s Hospital Colorado, Aurora, CO;; 19 Department of Biomedical Informatics, Columbia University, New York, NY;; 20 Health Management and Informatics, University of Missouri School of Medicine, Columbia, MO;; 21 Division of Epidemiology, Department of Medicine, Vanderbilt University Medical Center, Nashville, TN;; 22 Division of Advanced Pediatrics and Primary Care, Ann and Robert H. Lurie Children’s Hospital of Chicago, Chicago, IL;; 23 Division of Advanced General Pediatrics and Primary Care, Northwestern University Feinberg School of Medicine, Chicago, IL;; 24 Penn Medicine Center for Evidence-Based Practice (CEP), Philadelphia, PA;; 25 Penn Institute for Biomedical Informatics (IBI), Philadelphia, PA

## Abstract

**Objective:**

To evaluate the risks of incident dyslipidemia and abnormal body mass index (BMI) during the 28–179-day postacute phase after documented SARS-CoV-2 infection in a large pediatric sample.

**Study design:**

A retrospective cohort study using the Researching COVID to Enhance Recovery pediatric electronic health record datasets from 25 US children’s hospitals and health institutions, from March 2020 to September 2023. This study included 384 289 COVID-19-positive patients aged 0–21 years for dyslipidemia analyses and 285 559 aged 2–21 years for BMI analyses, each with at least 6 months of follow-up. COVID-19-negative controls included 1 080 413 and 817 315 patients, respectively. SARS-CoV-2 infection was defined by a positive polymerase chain reaction, antigen, or serologic test; a clinical diagnosis of COVID-19; or a documented diagnosis of post-acute sequelae of SARS-CoV-2. Incident dyslipidemia and abnormal BMI were identified using age-specific laboratory or anthropometric thresholds. Adjusted relative risks (aRRs) were estimated using propensity-score-stratified modified Poisson regression with multiple sensitivity analyses.

**Results:**

During the postacute phase, the COVID-19-positive cohort had higher rates of new-onset composite dyslipidemia (aRR 1.24; 95% CI 1.18–1.29) and abnormal BMI (aRR 1.15; 95% CI, 1.12–1.18). Results were robust to sensitivity and stratified analyses.

**Conclusions:**

Children and adolescents with documented COVID-19 infection were associated with an increased risk of new-onset dyslipidemia and abnormal BMI during the postacute phase, highlighting the need for metabolic monitoring after infection.

Children and adolescents infected with SARS-CoV-2 generally experience milder acute illness than adults, yet evidence suggests substantial postacute health consequences.^1^ Recent cohort studies have reported increased risks of cardiovascular, gastrointestinal, renal, and neuropsychiatric sequelae following pediatric COVID-19,^2–5^ suggesting that postacute sequelae extend beyond respiratory symptoms in children.

The metabolic health of children and adolescents represents a critical public health concern, particularly as childhood obesity and dyslipidemia are established risk factors for various health issues, such as coronary artery disease, metabolic dysfunction-associated steatosis liver disease, and type 2 diabetes.^6,7^ Prepandemic data from the National Health and Nutrition Examination Survey indicated that 21% of U.S. children and adolescents aged 6–19 had at least one abnormal cholesterol measure, and 32% had a high body mass index (BMI).^8^ Although lifestyle disruptions during the pandemic (eg, decreased physical activity, dietary changes) have contributed to rising rates of pediatric abnormal BMI and dyslipidemia, the extent to which these trends reflect direct postinfectious effects of COVID-19 remains uncertain. Recent studies found associations between preinfection BMI and postacute COVID-19 outcomes,^9^ yet whether COVID-19 infection is associated with subsequent abnormal BMI and dyslipidemia in children has not been comprehensively evaluated.

In adults, one study leveraging national health databases from the US Department of Veterans Affairs had found an increased risk of incident dyslipidemia in COVID-19 survivors, with effects persisting well beyond 1 year.^10^ Although emerging evidence suggests that children may also experience metabolic disturbances related to inflammatory responses to SARS-CoV-2,^11^ the pediatric data remain scarce: existing studies are often constrained by short follow-up or incomplete lipid measurements,^12^ leaving the metabolic consequences of COVID-19 in children and adolescents largely undefined. This knowledge gap is particularly concerning given that metabolic abnormalities acquired during childhood often persist into adulthood, potentially amplifying lifetime cardiovascular risk.^13,14^

We conducted a retrospective cohort study using the Researching COVID to Enhance Recovery (RECOVER) electronic health records (EHRs) database, involving 25 US children’s hospitals and health institutions, to explore whether documented SARS-CoV-2 infection was associated with increased risks of dyslipidemia and abnormal BMI among children and adolescents, during the postacute period (28–179 days). By analyzing 384 289 COVID-19-positive patients for dyslipidemia outcomes and 285 559 for BMI outcomes, with corresponding COVID-19-negative controls, this investigation represents the largest comprehensive assessment of post-COVID-19 metabolic sequelae in the pediatric population. Our findings aim to inform clinical monitoring strategies and preventive interventions for the millions of children recovering from COVID-19 worldwide.

## Methods

### Data Sources

This retrospective cohort study constitutes human subject research. Institute Review Board approval was obtained under Biomedical Research Alliance of New York (BRANY) protocol number 21-08-508. As part of the BRANY Institute Review Board process, the protocol has been reviewed in accordance with the Strengthening the Reporting of Observational Studies in Epidemiology reporting guideline.^15^ The BRANY waived the need for consent and HIPAA authorization. This study is affiliated with the NIH RECOVER Initiative (https://recovercovid.org/), a project dedicated to understanding the enduring impacts of COVID-19. The dataset comprises 25 contributing sites (listed in source of funding). Data were extracted from the RECOVER database version s10, collected till September 2023, more details in Supplementary Section S1A, B, available at www.jpeds.com).

### Cohort Construction

This retrospective cohort study drew on EHR records from March 2020 through September 2023 to assemble two parallel cohorts with at least 6 months of follow-up: (1) children and adolescents aged 0–21 years^16^ for dyslipidemia analysis, and (2) those aged 2–21 years for abnormal BMI analysis. For the COVID-19-positive group, the index date was defined as the earliest documented evidence of SARS-CoV-2 infection, a positive polymerase chain reaction, serology, antigen tests, or a documented diagnosis of COVID-19 or postacute sequelae of SARS-CoV-2–thereby anchoring follow-up at the actual onset of acute illness. We contrasted these patients with a COVID-19-negative control group with no record of SARS-CoV-2 infection who had at least one negative COVID-19 test during the same calendar period. For the control group, the index date was randomly assigned based on the empirical distribution of index dates in COVID-19 patients, to ensure comparable temporal risk windows. We excluded patient who did not have any clinical visits in the 24 months (729 days) to 7 days before their index date (baseline period) or between 28 and 179 days after their index date (postacute phase of COVID-19). In addition, patients with a history of dyslipidemia or abnormal BMI during the baseline period were further excluded from the corresponding analyses. For abnormal BMI study cohorts, we further excluded patients who took weight-modifying medications as well as those with associated abnormal conditions at the baseline period (refer to Supplementary Section S1C, available at www.jpeds.com for further details).

### Defining Dyslipidemia Outcomes

We adapted our lipid thresholds from Xu et al’s work^10^ and established pediatric guidelines.^14,17^ Dyslipidemia outcomes consisted of the following abnormal lipid laboratory results: abnormal total cholesterol: ≥ 200 mg/dL; abnormal triglycerides ≥ 100 mg/dL (ages 0–9 years); ≥ 130 mg/dL for (ages 10–19 years), ≥ 150 mg/dL for (ages 20–21 years); abnormal low-density lipoprotein (LDL) cholesterol: ≥ 130 mg/dL; abnormal high-density lipoprotein (HDL) cholesterol: < 40 mg/dL; abnormal non-HDL cholesterol: ≥ 145 mg/dL. We assessed incidents of each dyslipidemia outcome during the postacute phase of COVID-19 among those without any history of dyslipidemia in the 2 years preceding the index. The composite of any dyslipidemia outcome, “any abnormal lipid laboratory result”, defined as the first occurrence of any of the above thresholds during the study period.

### Defining Abnormal BMI Outcome

Abnormal BMI outcomes were determined based on age-appropriate BMI thresholds from recent pediatric growth and treatment guidelines.^18,19^ For ages 2–18 years: BMI z-score≥ 95th percentile, for ages 19–21 years: BMI ≥ 30 kg/m^2^. The incidence of abnormal BMI outcome was ascertained over the same postacute phase of COVID-19, excluding individuals with any history of BMI above the threshold during the 2-year baseline period.

### Covariates

We adjusted for a comprehensive set of baseline characteristics to control for confounding and ensure balanced comparison between SARS-CoV-2-exposed and unexposed children. The demographic characteristics included: patient age at cohort entry (years), sex (female/male), and race and ethnicity. Race and ethnicity were obtained from structured demographic fields in the EHR as recorded by participating health systems. Categories were standardized and listed alphabetically as: Asian American/Pacific Islander, Hispanic, Multiple, Non-Hispanic Black, Non-Hispanic White, and Other/Unknown. These variables were included to account for differences in social and structural determinants of health rather than biological factors. The temporal and site factors included the year-month of cohort entry (from March 2020 to September 2023) and the study site. The health care utilization measures included: the number of inpatient, outpatient, and emergency department visits; the number of unique medications or prescriptions (0, 1, 2, ≥3); and the number of COVID-19 negative tests at the baseline period (0, 1, 2, ≥3). Chronic comorbidities were assessed using the Pediatric Medical Complexity Algorithm (no chronic condition, noncomplex chronic condition, complex chronic condition), and a list of pre-existing chronic conditions (see Table S1, available at www.jpeds.com for more details).

For the dyslipidemia analysis, we further controlled for obesity status during the baseline period and for whether each patient completed corresponding lipid laboratory tests at the baseline.

### Statistical Analysis

To ensure only incident cases were captured, we first implemented a 24-month baseline washout period by excluding any patients with pre-existing abnormalities of lipid or BMI. We then compared the incidences of new-onset dyslipidemia or abnormal BMI outcomes between COVID-19-positive and negative control cohorts, by calculating the number of patients developing the specified outcome during the postacute window divided by the total number of patients in each cohort. Outcomes with an overall incidence below 0.1% were excluded a priori to prevent model instability.^20^ Distributions of preference scores,^21^ a transformation of propensity scores that accounts for prevalence differences between groups, were examined to assess empirical equipoise. To mitigate potential confounding due to imbalanced baseline between comparable groups, we estimated each patient’s propensity score, the probability of being in the COVID-19-positive group given covariates, using a multivariable logistic regression model that included prespecified covariates as the independent variables. Patients were then stratified into six propensity-score strata, and within each stratum, we modeled each outcome via modified Poisson regression with robust variance, incorporating the stratum identifier as a covariate, to estimate adjusted relative risk (aRRs) to quantify the overall risk.^22^ The balance was assessed via standardized mean difference for each covariate. A difference of ≤ 0.1 was considered indicative of acceptable balance^23^ (see Supplementary Section S2A, Figure S1, available at www.jpeds.com for more details). All analyses were performed using R version 4.4.0 (R Foundation for Statistical Computing).

### Sensitivity Analysis

Recognizing that unmeasured factors may still bias our aRR estimates, we performed two complementary sensitivity checks. First, we conducted empirical calibration using 36 negative control outcomes (NCOs). NCOs were known *a priori* not to be causally associated with COVID-19, thereby quantifying and correcting any residual systematic error in the risk estimate. Second, for the dyslipidemia analysis, we stratified by baseline obesity status to assess potential differential effects. (See Supplementary Section S2B, C, available at www.jpeds.com).

## Results

### Cohort Identification

**Figure 1.**
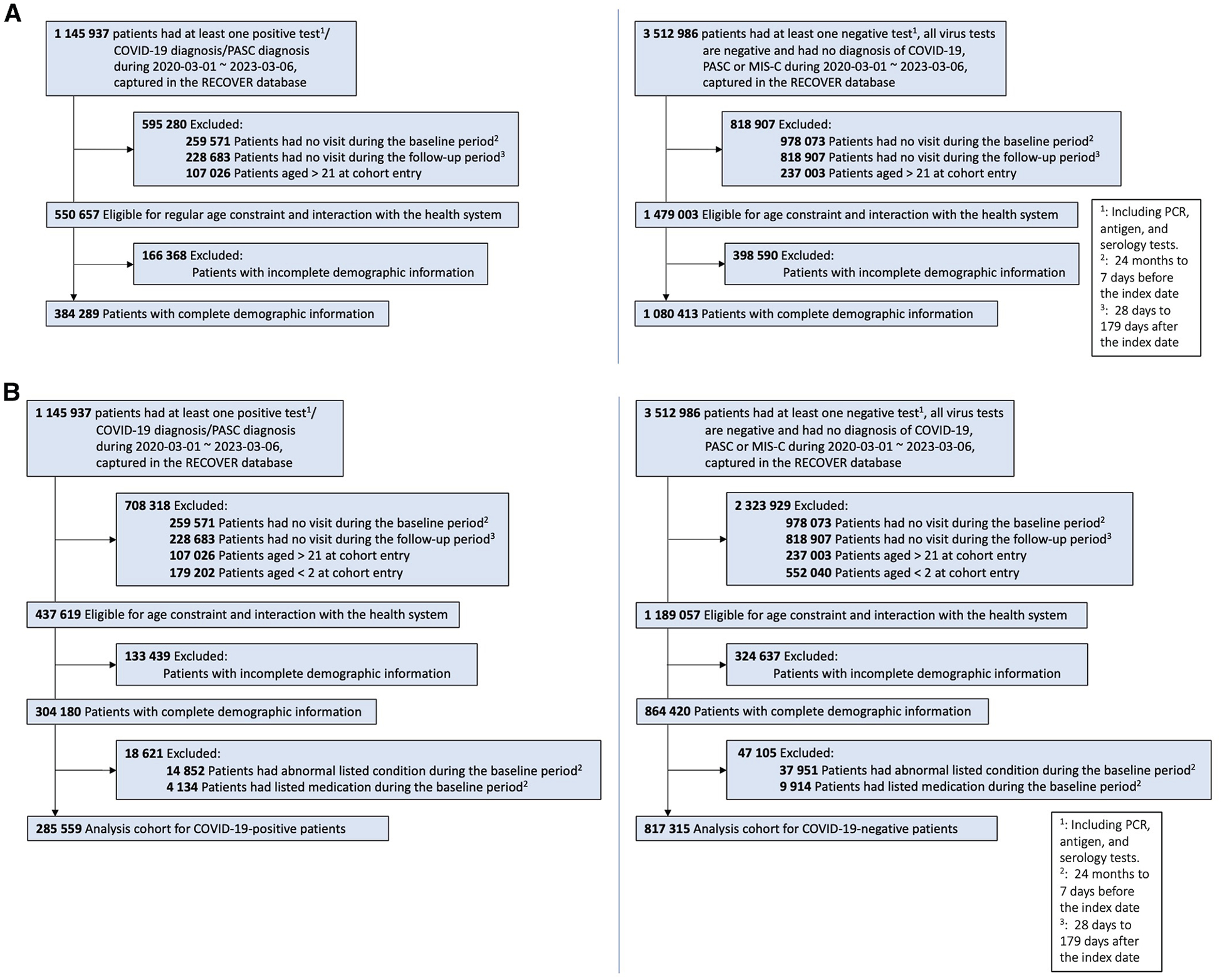
Cohort attrition diagrams for dyslipidemia and abnormal BMI study cohorts. **A,** Flowchart outlining sample selection for the dyslipidemia cohort, including COVID-19-positive and COVID-19-negative participants; **B,** Flowchart outlining sample selection for the abnormal BMI cohort, including COVID-19-positive and COVID-19-negative participants.

We included 384 289 COVID-19-positive children and adolescents for the dyslipidemia analysis (mean [SD] entry age, 8.6 [6.5] years) and 285 559 for the abnormal BMI analysis (10.5 [5.5] years). The COVID-19-negative control cohorts comprised 1 080 413 patients (7.9 [6.2] years) for dyslipidemia and 817 315 patients (9.5 [5.5] years) for abnormal BMI. Table I summarizes baseline characteristics, including race and ethnicity distributions using standardized, alphabetically ordered categories. Figure 1 illustrates an overview of the participant selection process for both COVID-19-positive and COVID-19-negative patients with eligible criteria in the dataset.

### Incidence of Postacute Abnormal Outcomes for Both COVID-19 and Control Groups

The incidence of postacute composite dyslipidemia (“any abnormal lipid result”) was 0.945% in the COVID-19-positive group compared with 0.691% in COVID-19-negative controls (Table II). Across individual lipid measures, incidence in the COVID-19-positive group ranged from 0.197% for LDL abnormalities to 0.576% for low HDL, compared with 0.149% and 0.414% in the COVID-19-negative group, respectively. For abnormal BMI, postacute incidence was 5.954% among those with prior COVID-19 vs 4.743% in controls.

### aRR of Postacute Abnormal Outcomes

**Figure 2.**
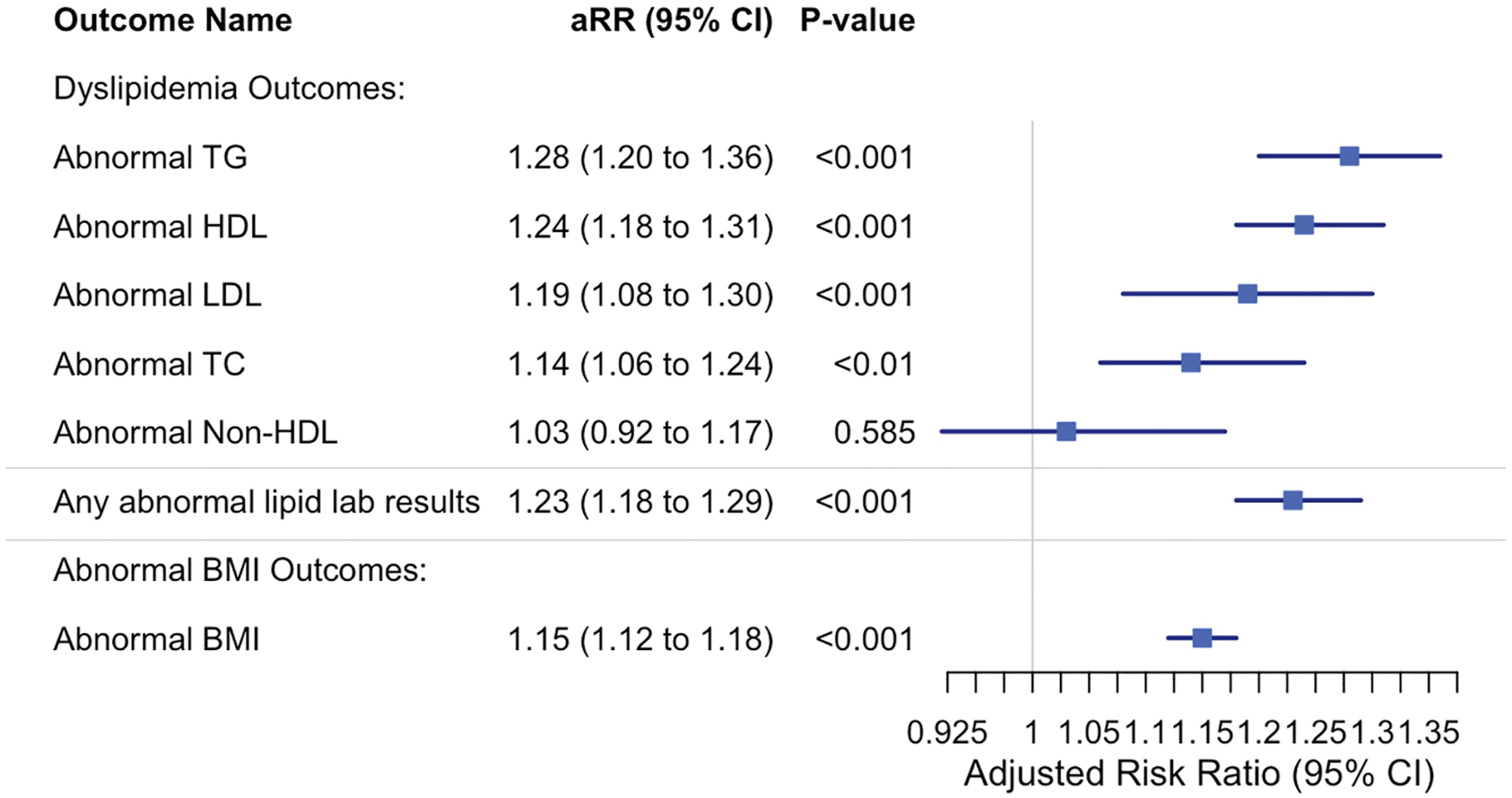
Adjusted relative risks of postacute dyslipidemia and abnormal BMI outcomes in COVID-19-positive vs COVID-19-negative cohorts. Forest plot displaying adjusted relative risks (aRRs) and 95% confidence intervals for incident postacute dyslipidemia outcomes and abnormal BMI. Estimates were obtained using modified Poisson regression models adjusted for demographic and clinical covariates. †Definitions and abbreviations: *Abnormal BMI*, BMI z-score≥ 95th percentile for ages 2–18 years, BMI ≥ 30 kg/m2 for ages 19–21 years; *Abnormal HDL cholesterol*, HDL cholesterol: < 40 mg/dL; *Abnormal LDL cholesterol*: LDL cholesterol: ≥ 130 mg/dL; *Abnormal non-HDL cholesterol*: non-HDL cholesterol: ≥ 145 mg/dL; *Abnormal TC*, total cholesterol (TC): ≥ 200 mg/dL; *Abnormal TG*, triglycerides (TG) ≥ 100 mg/dL for ages 0–9 years, ≥ 130 mg/dL for ages 10–19 years, ≥ 150 mg/dL for ages 20–21 years. Any abnormal lipid lab results: the first occurrence of any of the above thresholds during the study period.

After propensity score stratification, the documented SARS-CoV-2 infection was significantly associated with higher risks of most lipid abnormalities at the postacute phase (Figure 2). The outcomes, listed in decreasing order of magnitude, were: abnormal triglyceride levels (aRR 1.28, 95% CI 1.20–1.36; *P* < .001), abnormal HDL cholesterol levels (aRR 1.24, 95% CI 1.18–1.31; *P* < .001), abnormal LDL cholesterol levels (aRR 1.19, 95% CI 1.08–1.30; *P* < .001), total cholesterol levels (aRR 1.14, 95% CI 1.06–1.24; *P* < .01), and non-HDL cholesterol levels showed a nonsignificant trend (aRR 1.03, 95% CI 0.92–1.17; *P* = .585). The composite of any abnormal lipid result outcome yielded an aRR of 1.23 (95% CI 1.18–1.29; *P* < .001). For abnormal BMI outcome, the aRR was 1.15 (95% CI 1.12–1.18; *P* < .001).

### Sensitivity Analysis

Empirical calibration using 36 NCOs produced point estimates very close to the primary aRRs but with modestly wider confidence intervals, suggesting minimal residual bias (Table S2, available at www.jpeds.com). Stratification by baseline obesity status yielded consistent aRRs across subgroups, indicating that pre-existing adiposity did not materially modify our primary findings (Table S3, available at www.jpeds.com).

## Discussion

By leveraging 25 longitudinal EHR datasets, we found that children and adolescents with documented COVID-19 infection were associated with modestly increased risks of incident dyslipidemia and abnormal BMI among children and adolescents during the 28 to 179-day postacute phase. These findings represent the first large-scale evidence of post-COVID metabolic associations in the pediatric population, underscoring that children and adolescents may experience persistent metabolic consequences despite typically milder acute illness.

Our results extend observations from adult studies^10,24,25^ to the younger age group. The Veterans Affairs cohort study has shown an increased risk of incident dyslipidemia in adult COVID-19 survivors, with hazard ratios ranging from 1.2 to 1.27 for different lipid abnormalities.^10^ Our pediatric findings demonstrate lower but predominantly significant effect sizes (aRR ranging from 1.04 to 1.28), suggesting that although children may have protective factors, they remain susceptible to dyslipidemia sequelae of SARS-CoV-2 infection. Similar patterns emerged for abnormal BMI associations. These BMI findings align with documented pandemic-related weight gain in children,^26^ though our results suggest that COVID-19 infection itself may contribute additional metabolic risk beyond broader pandemic lifestyle effects.

Several mechanisms may explain these associations. SARS-CoV-2 may disrupt lipid homeostasis either through angiotensin-converting enzyme 2 receptor-mediated signaling^27^ or directly via its spike protein, both of which contribute to the upregulation of lipid synthesis pathways and metabolic dysregulation. Infection also triggers systemic inflammation, notably elevations in interleukin-6 and tumor necrosis factor-alpha, which have been associated with acute dyslipidemia, including reduced HDL cholesterol and elevated triglycerides,^28,29^ likely through hepatic lipid metabolism disruption.

Although individually modest, our findings may have substantial population-level implications given widespread pediatric COVID-19 exposure. Childhood metabolic abnormalities often persist into adulthood, potentially amplifying lifetime cardiovascular risk.^6,7,13,14^ These considerations support incorporating enhanced metabolic monitoring into post-COVID pediatric follow-up care.

This study has several strengths. First, it leveraged the RECOVER EHR consortium across 25 U S. children’s hospitals to assemble the largest and most heterogeneous cohort to date for examining postacute abnormalities of lipid and BMI in youth. Second, inclusion of contemporaneous, COVID-19-negative controls and rigorous propensity-score stratification across more than hundreds of covariates minimized confounding. Third, the use of laboratory-based definitions for lipid abnormalities and objectively measured BMI reduced misclassification compared with diagnosis codes alone. Fourth, multiple sensitivity analyses, including empirical calibration with 36 NCOs and stratified analyses by baseline obesity, further reinforced the reliability of our results. Finally, consistent effect estimates across individual lipid subtypes and composite outcomes underscore the clinical relevance of our findings.

However, several limitations merit consideration. First, documented SARS-CoV-2 infections may be underascertained if testing occurred outside the captured EHR network or among asymptomatic children, while minor differential misclassification could be corrected.^30^ Second, although we balanced over hundreds of covariates, unmeasured factors, such as diet, physical activity, sleep patterns, and socioeconomic status may still confound the associations; our NCO calibration provides some correction, but residual bias cannot be excluded. Third, indication bias may also be present because lipid assessments are not routinely conducted in pediatric practice; to address this, we further included a baseline indicator for participating in lipid tests in our covariates set (see Supplementary Section S1E, available at www.jpeds.com for more details). All confounders were balanced after propensity score stratification, with an standardized mean difference of less than 0.1 (Figure S1, available at www.jpeds.com). Furthermore, we excluded children receiving COVID-specific treatments but could not fully account for other medications (eg, corticosteroids) that influence metabolic outcomes. Our analysis focused on early postacute changes (28–179 days), leaving longer-term trajectories unclear. Finally, as an observational study, causality cannot be definitively established, although the consistency across sensitivity analyses strengthens our inference that SARS-CoV-2 infection contributes to early dyslipidemia and BMI abnormalities. ■

## Supplementary Material

1

2

3

4

5

## Figures and Tables

**Table I.** Baseline characteristics of children and adolescents in the dyslipidemia and abnormal BMI study cohorts

(a) Dyslipidemia study cohorts	COVID-19-positive (n = 384 289)	COVID-19-negative (n = 1 080 413)
Age at entry (years)		
<1	48 984 (12.7%)	117 925 (10.9%)
1–4	88 740 (23.1%)	301 107 (27.9%)
5–11	104 349 (27.2%)	322 470 (29.8%)
12–18	115 255 (30.0%)	283 705 (26.3%)
19–21	26 961 (7.0%)	55 206 (5.1%)
Sex		
Female	192 471 (50.1%)	523 618 (48.5%)
Male	191 818 (49.9%)	556 795 (51.5%)
Race/ethnicity		
AAPI	18 185 (4.7%)	52 703 (4.9%)
NHB	64 265 (16.7%)	172 656 (16.0%)
Hispanic	88 231 (23.0%)	231 539 (21.4%)
Multiple	9057 (2.4%)	31 407 (2.9%)
Other/unknown	32 665 (8.5%)	95 676 (8.9%)
NHW	171 886 (44.7%)	496 432 (45.9%)
Health system		
A	28 319 (7.4%)	91 745 (8.5%)
B	54 727 (14.2%)	141 342 (13.1%)
C	15 557 (4.0%)	66 974 (6.2%)
D	5543 (1.4%)	15 538 (1.4%)
E	8428 (2.2%)	39 785 (3.7%)
F	13 281 (3.5%)	51 177 (4.7%)
G	7432 (1.9%)	17 770 (1.6%)
H	9946 (2.6%)	30 753 (2.8%)
I	5630 (1.5%)	15 006 (1.4%)
J	23 862 (6.2%)	108 503 (10.0%)
K	2310 (0.6%)	4811 (0.4%)
L	25 258 (6.6%)	90 365 (8.4%)
M	18 780 (4.9%)	44 484 (4.1%)
N	9881 (2.6%)	32 501 (3.0%)
O	55 869 (14.5%)	117 756 (10.9%)
P	16 548 (4.3%)	25 802 (2.4%)
Q	2902 (0.8%)	5589 (0.5%)
R	25 707 (6.7%)	13 244 (1.2%)
S	5352 (1.4%)	16 450 (1.5%)
T	4971 (1.3%)	27 325 (2.5%)
U	12 895 (3.4%)	39 450 (3.7%)
V	1134 (0.3%)	1873 (0.2%)
W	11 776 (3.1%)	37 278 (3.5%)
X	12 755 (3.3%)	31 826 (2.9%)
Y	5426 (1.4%)	13 066 (1.2%)
Cohort entry period (month/year)		
03/2020–05/2020	3772 (1.0%)	20 157 (1.9%)
06/2020–08/2020	12 050 (3.1%)	71 838 (6.6%)
09/2020–11/2020	21 443 (5.6%)	95 570 (8.8%)
12/2020–02/2021	36 568 (9.5%)	93 551 (8.7%)
03/2021–05/2021	21 898 (5.7%)	98 211 (9.1%)
06/2021–08/2021	21 659 (5.6%)	96 832 (9.0%)
09/2021–11/2021	35 952 (9.4%)	145 716 (13.5%)
12/2021–02/2022	111 912 (29.1%)	111 129 (10.3%)
03/2022–05/2022	28 759 (7.5%)	84 261 (7.8%)
06/2022–08/2022	43 389 (11.3%)	66 174 (6.1%)
09/2022–11/2022	22 585 (5.9%)	105 954 (9.8%)
12/2022–03/2023	24 302 (6.3%)	91 020 (8.4%)
Obesity		
No	201 393 (52.4%)	631 207 (58.4%)
Yes	151 562 (39.4%)	346 113 (32.0%)
Unknown	31 334 (8.2%)	103 093 (9.5%)
PMCA		
No chronic condition	265 500 (69.1%)	744 977 (69.0%)
Noncomplex chronic condition	64 989 (16.9%)	181 753 (16.8%)
Complex chronic condition	53 800 (14.0%)	153 683 (14.2%)
Number of negative COVID-19 tests		
0	226 376 (58.9%)	797 816 (73.8%)
1	82 257 (21.4%)	177 513 (16.4%)
≥2	75 656 (19.7%)	105 084 (9.7%)
(b) Abnormal BMI study cohort	COVID-19-positive (n = 285 559)	COVID-19-negative (n = 817 315)
Age at entry (years)		
2–4	55 502 (19.4%)	197 319 (24.1%)
5–11	100 619 (35.2%)	312 416 (38.2%)
12–18	106 539 (37.3%)	261 079 (31.9%)
19–21	22 899 (8.0%)	46 501 (5.7%)
Sex		
Female	144 438 (50.6%)	401 227 (49.1%)
Male	141 121 (49.4%)	416 088 (50.9%)
Race/ethnicity		
AAPI	47 093 (16.5%)	127 420 (15.6%)
NHB	65 197 (22.8%)	177 705 (21.7%)
Multiple	6217 (2.2%)	22 433 (2.7%)
Other/unknown	23 178 (8.1%)	70 353 (8.6%)
White	130 337 (45.6%)	378 923 (46.4%)
NHW	13 537 (4.7%)	40 481 (5.0%)
Health system		
A	9292 (3.3%)	22 343 (2.7%)
B	14 602 (5.1%)	36 204 (4.4%)
C	1831 (0.6%)	3634 (0.4%)
D	3621 (1.3%)	9356 (1.1%)
E	5235 (1.8%)	28 124 (3.4%)
F	820 (0.3%)	1361 (0.2%)
G	10 279 (3.6%)	40 418 (4.9%)
H	9542 (3.3%)	29 799 (3.6%)
I	17 014 (6.0%)	79 930 (9.8%)
J	3688 (1.3%)	12 079 (1.5%)
K	4044 (1.4%)	11 553 (1.4%)
L	18 209 (6.4%)	68 254 (8.4%)
M	6051 (2.1%)	14 334 (1.8%)
N	10 514 (3.7%)	49 762 (6.1%)
O	2447 (0.9%)	4489 (0.5%)
P	7090 (2.5%)	23 959 (2.9%)
Q	18 654 (6.5%)	8651 (1.1%)
R	40 943 (14.3%)	105 320 (12.9%)
S	8502 (3.0%)	27 874 (3.4%)
T	7091 (2.5%)	24 996 (3.1%)
U	21 157 (7.4%)	69 069 (8.5%)
V	13 101 (4.6%)	19 090 (2.3%)
W	3469 (1.2%)	10 735 (1.3%)
X	3084 (1.1%)	19 393 (2.4%)
Y	45 279 (15.9%)	96 588 (11.8%)
Cohort entry period (month/year)		
03/2020–05/2020	2618 (0.9%)	13 956 (1.7%)
06/2020–08/2020	9882 (3.5%)	55 685 (6.8%)
09/2020–11/2020	18 107 (6.3%)	76 894 (9.4%)
12/2020–02/2021	29 980 (10.5%)	74 372 (9.1%)
03/2021–05/2021	17 894 (6.3%)	77 687 (9.5%)
06/2021–08/2021	17 584 (6.2%)	73 980 (9.1%)
09/2021–11/2021	29 346 (10.3%)	116 210 (14.2%)
12/2021–02/2022	83 019 (29.1%)	83 411 (10.2%)
03/2022–05/2022	20 592 (7.2%)	62 506 (7.6%)
06/2022–08/2022	27 685 (9.7%)	45 275 (5.5%)
09/2022–11/2022	14 584 (5.1%)	74 668 (9.1%)
12/2022–03/2023	14 268 (5.0%)	62 671 (7.7%)
PMCA		
No chronic condition	195 349 (68.4%)	558 638 (68.4%)
Noncomplex chronic condition	35 445 (12.4%)	105 438 (12.9%)
Complex chronic condition	54 765 (19.2%)	153 239 (18.7%)
Number of negative COVID-19 tests		
0	169 916 (59.5%)	610 009 (74.6%)
1	61 672 (21.6%)	133 006 (16.3%)
≥2	53 971 (18.9%)	74 300 (9.1%)

*AAPI*, Asian American and Pacific Islander; *NHB*, non-Hispanic Black; *NHW*, non-Hispanic White; *PMCA*, Pediatric Medical Complexity Algorithm.

(a) Shows characteristics of the dyslipidemia cohort and (b) shows characteristics of the abnormal BMI cohort for COVID-19-positive and COVID-19-negative groups, separately. Health system names were deidentified and represented as letters (A-Y) in accordance with data use agreements and to protect site confidentiality.

**Table II.** Raw postacute incidence of dyslipidemia and abnormal BMI outcomes in COVID-19-positive and COVID-19-negative cohorts

Outcome name	COVID-19-positive (n = 384 289)	COVID-19-negative (n = 1 080 413)
Dyslipidemia outcomes		
Abnormal TG	0.491% (1851/376 655)	0.342% (3639/1 064 911)
Abnormal HDL cholesterol	0.576% (2164/375 603)	0.414% (4395/1 061 721)
Abnormal LDL cholesterol	0.197% (751/381 401)	0.149% (1598/1 074 385)
Abnormal TC	0.268% (1020/380 069)	0.211% (2260/1 071 771)
Abnormal non-HDL cholesterol	0.111% (425/382 449)	0.088% (946/1 077 298)
Composite dyslipidemia outcomes		
Any abnormal lipid lab results	0.945% (3490/369 33)	0.691% (7254/1 049 067)
Abnormal BMI outcomes	COVID-19-positive (n = 285 559)	COVID-19-negative (n = 817 315)
Abnormal BMI	5.954% (10 689/179 518)	4.743% (27 967/589 622)

Raw incidence (in %), calculated as the absolute number of patients with outcome during the postacute phase divided by the number of total patients that did not have the specific outcome during the baseline period. †Definitions and abbreviations: *Abnormal BMI*, BMI z-score≥ 95th percentile for ages 2–18 years, BMI ≥ 30 kg/m2 for ages 19–21 years; *Abnormal HDL cholesterol*, HDL cholesterol: < 40 mg/dL; *Abnormal LDL cholesterol*, LDL cholesterol: ≥ 130 mg/dL; Abnormal non-HDL cholesterol, non-HDL cholesterol: ≥ 145 mg/dL; *Abnormal TC*, total cholesterol (TC): ≥ 200 mg/dL; *Abnormal TG*, triglycerides (TG) ≥ 100 mg/dL for ages 0–9 years, ≥ 130 mg/dL for ages 10–19 years, ≥ 150 mg/dL for ages 20–21 years. Any abnormal lipid lab results: the first occurrence of any of the above thresholds during the study period.

## Abbreviations

BMI: Body mass index
BRANY: Biomedical Research Alliance of New York
EHR: Electronic health record
HDL: High-density lipoprotein
LDL: Low-density lipoprotein
NCO: Negative control outcome
RECOVER: Researching COVID to Enhance Recovery
