# Supplementary material for: Post-Acute Dyslipidemia and Abnormal Body Mass Index in Children and Adolescents with COVID-19: A Cohort Study from the RECOVER Initiative
Source: J Pediatr. Author manuscript; Available in PMC 2026 May 30. (PMC13221949; doi:10.1016/j.jpeds.2026.114996)
Supplement: 1 [file NIHMS2168194-supplement-1.docx]

Supplementary Appendix to:

Post-acute dyslipidemia and abnormal BMI in children and adolescents with COVID-19: An EHR Cohort Study from the RECOVER Initiative

Table of Contents

[Section S1 Supplemental Methods 2](#_Toc215604083)

[A. Description of electronic health records (EHR) data 2](#_Toc215604084)

[B. RECOVER population and generalizability 2](#_Toc215604085)

[C. Cohort definition and observation windows 3](#_Toc215604086)

[D. Propensity-score (PS) models and stratification 5](#_Toc215604087)

[E. Indication Bias for Dyslipidemia Analysis 6](#_Toc215604088)

[F. Empirical equipoise assessment 6](#_Toc215604089)

[Section S2 Supplemental Results: 8](#_Toc215604090)

[A. Patient characteristic balance in primary analysis 8](#_Toc215604091)

[B. Sensitivity Analyses: Negative Control Experiments 8](#_Toc215604092)

[C. Stratification Analyses: Obesity Status Subgroups 10](#_Toc215604093)

[D. Additional Sensitivity Analyses: Offset-Term Adjustment and IPTW Analysis 12](#_Toc215604094)

[Section S3 Supplemental Appendix: RECOVER Consortium Group Group Members 13](#_Toc215604095)

[Reference 15](#_Toc215604096)

# Section S1 Supplemental Methods

## A. Description of electronic health records (EHR) data

The real-world data utilized in our analysis is derived from electronic health records (EHRs), covering a wide range of healthcare interaction information routinely collected and stored by hospitals. This includes clinical data such as diagnoses and treatments, laboratory and test results, and administrative data including patient demographics and billing information. The hospital-based EHR data from the Researching COVID to Enhance Recovery (RECOVER) Initiative COVID-19 Database served as the basis for defining and determining exposure, outcomes, and covariates. Unlike General Practitioner (GP) data, self-reported data, or external data sources, our study used the structured, standardized EHR entries made by healthcare providers within hospital settings. EHR data provides a more detailed and integrated view of a patient's health status, medical history, and healthcare interactions across various providers and settings.

## B. RECOVER population and generalizability

The National Institutions of Health (NIH) launched the new RECOVER initiative in 2021 to leverage electronic health record (EHR) data to better identify and characterize patients with post-acute sequelae of SARS-CoV-2 infection (PASC). RECOVER obtains EHRs from three large national healthcare networks within the United States, covering regional catchment areas across 41 states. These networks collectively hold the EHRs of over 60 million patients, including records from more than 7 million individuals who have been affected by COVID-19. RECOVER collaborates with the National Institutes of Health's (NIH) All of Us Research Program, which contributes additional health records to this vast database. Together, these sources comprise one of the world's largest collections of EHRs.

The dataset comprises information from 25 contributing sites, listed in **Source of Funding** section. RECOVER-EHR Consortium Members list attached in **Section S3 Supplemental Appendix**.

## C. Cohort definition and observation windows

We assembled two parallel pediatric cohorts (dyslipidemia and abnormal BMI) from March 2020 through September 2023. Entry criteria and observation windows were defined as follows:

- **Age at Index**
  - Dyslipidemia cohorts: 0-21 years
  - Abnormal-BMI cohorts: 2-21 years
- **Index Date**
  - **COVID-19 Positive Cohort**: Earliest evidence of SARS-CoV-2 infection: positive PCR, antigen, or serology test; or documented diagnosis of COVID-19 or post-acute sequelae of SARS-CoV-2 (PASC)
  - **COVID-19 Negative Control Cohort**: No record of SARS-CoV-2 infection, ≥1 negative COVID-19 test; index date randomly sampled from the empirical distribution of infection dates in COVID-19 positive cohort
- **Outcome of interests**:
  - **Dyslipidemia**:
    - Abnormal TC: Total cholesterol (TC) ≥ 200 mg/dL
    - Abnormal TG:
      - Triglycerides (TG) ≥ 100 mg/dL (ages 0-9 years)
      - ≥ 130 mg/dL for (ages 10-19 years)
      - ≥ 150 mg/dL for (ages 20-21 years)
    - Abnormal LDL: Low-density lipoprotein (LDL) cholesterol ≥ 130 mg/dL
    - Abnormal HDL: High-density lipoprotein (HDL) cholesterol < 40 mg/dL
    - Abnormal non-HDL: Non-HDL cholesterol ≥ 145 mg/dL

Note: we removed Abnormal Apolipoprotein B: Apolipoprotein B ≥ 110 mg/dL due to an overall incidence below 0.1%.

- - Abnormal BMI:
    - - BMI z-score≥ 95th percentile (ages 2-18 years)
      - BMI ≥ 30 kg/m^2^ (ages 19-21 years)
- **Baseline Period (washout & covariate capture)**
  - Window: 7-729 days before index
  - Required ≥1 clinical encounter (inpatient, outpatient, or ED)
  - Excluded if any record of the outcome of interest during this period
  - **Additional Exclusions (Abnormal-BMI cohorts only)**:
    - Pre-existing abnormal conditions:
      - Cancer, cystic fibrosis, eating disorder, sickle cell disease, Crohn’s disease, ulcerative colitis, HIV, growth hormone deficiency, Cushing syndrome, panhypopituitarism, BMI less then 5th percentile for age and sex, pregnancy, bariatric surgery
    - Weight-modifying medications:
      - metformin, orlistat, liraglutide, exenatide, dulaglutide, semaglutide, setmelanotide, phentermine, topiramate
- **Follow-Up Period (outcome ascertainment, post-acute phase)**
  - Window: 28-179 days after index
  - Required ≥1 clinical encounter

To guarantee a comprehensive follow-up for all participants, we mandated that entry into the cohort, applicable to both the exposure and control groups, be timed no later than 179 days prior to the conclusion of the study period. This stipulation was critical to secure a full 179-day follow-up duration for each participant, thereby upholding the integrity of the follow-up data.

## D. Propensity-score (PS) models and stratification

We fitted a large-scale PS model for the study cohort with baseline patient characteristics including

- Demographics (age at index date; sex; race and ethnicity)
- Obesity (Yes/No/Unknown)
- Chronic condition indicator as defined by the Pediatric Medical Complexity Algorithm (PMCA)
  - No chronic condition (PMCA = 0)
  - Non-complex chronic condition (PMCA = 1)
  - Complex chronic condition comorbidities (PMCA = 2)
- The existence of a list of 205 chronic conditions 24 months ~ 7 days prior to the index
- Healthcare utilization 24 months ~ 7 days prior to index date categorized to 0,1,2, ≥3
  - Number of inpatient visits
  - Number of outpatient visits
  - Number of emergency department (ED) visits
  - Number of unique medications
  - Number of negative COVID-19 tests
- Cohort entry date (index date) categorized to 1 month
- Healthcare system index

To summarize baseline adjustment, we applied propensity-score stratification to improve robustness in larger EHR cohort. The patients were then stratified into 6 equally spaced PS strata. Stratification offers a straightforward and transparent way to compare patients within comparable PS ranges and is well suited for large EHR cohorts. To complement this approach, we also conducted IPTW as an additional sensitivity analysis, with results summarized narratively in [D. Additional Sensitivity Analyses: Offset-Term Adjustment and IPTW Analysis](#_D._Additional_Sensitivity).

## E. Indication Bias for Dyslipidemia Analysis

Lipid testing is not routinely conducted in pediatric practice, children who underwent these evaluations may differ systematically from those who did not. For example, patients who are more likely to complete the lipid lab test may also be more likely to have underlying health conditions that predispose them to abnormal lipid levels. Completing the lipid lab test may be associated with both exposure (COVID-19 infected or not) and outcome (abnormal lipid lab results). To address this, we further included a baseline indicator for participating lipid test in our covariates set^5,6^. All confounders were balanced after propensity score stratification, with an SMD of less than 0.1. **Figure S1** shows two representative outcomes, with similar balance observed for all remaining outcomes.

## F. Empirical equipoise assessment

To assess the similarity across study groups, we present the preference score. This metric refines the propensity score by integrating treatment prevalence, facilitating an easily understood comparison. The preference score (F) is mathematically derived from the propensity score (S) and the treatment prevalence (P) using the following formula^7,8^:

$$\ln\left( \frac{F}{1-F} \right)=\ln\left( \frac{S}{1-S} \right)-\ln\left( \frac{P}{1-P} \right).$$

Preference score distributions for COVID-19-positive and COVID-19-negative participants were examined for each primary outcome of interested. Across outcomes, 63.4% to 67.0% of participants fell within the region of empirical equipoise, indicating substantial overlap and suggesting adequate comparability between exposure groups.

# Section S2 Supplemental Results:

## A. Patient characteristic balance in primary analysis

We evaluated covariate balance using standardized mean differences (SMDs). **Figure S1** displays the balance diagnostics before and after propensity score stratification for two representative outcomes. In both cases, stratification substantially reduced covariate imbalance, with all post-stratification SMDs (purple triangles) falling below the conventional threshold of 0.1, demonstrating adequate covariate balance. The remaining outcomes demonstrated similar patterns of improved balance.

## B. Sensitivity Analyses: Negative Control Experiments

To evaluate the robustness of our findings, we conducted a series of negative control outcome (NCO) experiments using a predefined set of 36 outcomes. Negative control outcomes were defined as clinical conditions that are not plausibly causally related to the exposure of interest (COVID-19 in our analyses). The outcome list was developed in collaboration with pediatric clinicians based on domain knowledge and clinical judgment. Specifically, the list comprised acne; astigmatism; autism or autistic disorder; closed fracture of distal end of radius; closed injury of head; concussion; contact dermatitis; diaper rash; displacements of bone; epilepsy; falls; foreign body in ear; impetigo; inguinal hernia; injury of finger; injury of free lower limb; injury of head; injury of left leg; injury of right foot; injury of right hand; injury of right leg; injury of upper extremity; insect bite; myopia; plagiocephaly; scoliosis; seizure; snoring or obstructive sleep apnea; speech delay; speech dysfunction; sprain of ankle; tinea capitis; tinea corporis; tongue tie; umbilical hernia; and wax in ear or impacted cerumen.

We employed a set of 36 NCOs to assess the presence of residual bias and support calibration of our estimated risk ratios (RRs). These outcomes were selected based on clinical judgment by two board-certified pediatricians (DT, CF) and were defined as conditions with no known or plausible causal association with SARS-CoV-2 infection. Under the null hypothesis, the exposure is not expected to influence the incidence of these outcomes.

To implement negative control calibration, we first estimated the empirical null distribution derived from the effect estimates of the negative control outcomes. This distribution was then used to adjust the RRs from the primary analysis to account for potential systematic error. The methodological framework for deriving and applying the empirical null follows the principles outlined by Schuemie et al.^9-11^.

To improve the stability of these estimates and avoid excessive uncertainty, negative control outcomes with a cohort-wide incidence below 0.1% were excluded from the calibration analysis. We quantified the magnitude of systematic error using the expected absolute systematic error (EASE), calculated as the absolute difference between the log-transformed observed RR and the (assumed null) true RR. The EASEs across all analyese were ranging from 0.13 to 0.15. A minimal degree of systematic bias was detected, and the calibrated estimates are reported in **Table S2**. The confidence intervals are wider than in the primary results, as expected under calibration, but the point estimates maintain the same overall direction and trend.

## C. Stratification Analyses: Obesity Status Subgroups

We conducted subgroup analyses for dyslipidemia outcomes stratified by baseline obesity status using age- and sex-specific BMI percentiles for individuals aged 0-19 years and standard BMI thresholds for those aged 20-21 years. Obesity categories were defined as follows:

- **Healthy weight**:
  - For participants aged 0-19 years: BMI z-score between –1.645 and < 1.036 (corresponding to the 5th to <85th percentiles)
  - For participants aged 20-21 years: BMI between 18.5 and <25 kg/m²
- **Obesity class 1**:
  - For participants aged 0-19 years: BMI z-score > 1.645 (≥95th percentile)
  - For participants aged 20-21 years: BMI between 30 and <35 kg/m²
- **Obesity class 2**:
  - For participants aged 0-19 years: BMI between 120% and <140% of the 95th percentile for age and sex
  - For participants aged 20-21 years: BMI between 35 and <40 kg/m²
- **Obesity class 3**:
  - For participants aged 0-19 years: BMI ≥140% of the 95th percentile for age and sex or absolute BMI ≥40 kg/m²
  - For participants aged 20-21 years: BMI ≥40 kg/m²

Across weight categories, **Table S3** shows that the direction and magnitude of the associations between COVID-19 and post-acute dyslipidemia outcomes were broadly similar, with modest variation in adjusted relative risks. Wider confidence intervals in smaller subgroups primarily reflect reduced sample size rather than largely different associations.

## D. Additional Sensitivity Analyses: Offset-Term Adjustment and IPTW Analysis

**Offset Term Sensitivity Analysis.**

To address concerns about potential variation in follow-up time, we refitted all modified Poisson models with the number of follow-up days included as an offset term. Because the outcomes were defined as binary indicators of whether a new dyslipidemia or abnormal BMI event occurred within the fixed 28-179-day post-acute window, all participants were observed over the same timeframe, and an offset term was not required for the primary analysis.

The offset models nevertheless produced results that were highly consistent with the primary estimates. The adjusted relative risks differed only at the second decimal place, and no conclusions regarding the direction or statistical significance of the associations changed. Overall, incorporating follow-up days as an offset confirmed the robustness of the primary findings.

**IPTW Analysis.**

We additionally estimated adjusted risks using inverse probability of treatment weighting. IPTW results were directionally consistent with the primary propensity-score-stratified estimates, with similar magnitudes across all dyslipidemia and BMI outcomes.

# Section S3 Supplemental Appendix: RECOVER Consortium Group Group Members

**RECOVER-EHR Consortium Members**

**Study Group Leads**

Ivan Diaz

[ivan.diaz@nyulangone.org](mailto:ivan.diaz@nyulangone.org)

Rachel Kenny

[Rachel.kenny@nyulangone.org](mailto:Rachel.kenny@nyulangone.org)

**EHR Clinical Science Core**

**New York University**

*Iván Diaz, PI*

*Rachel Kenny, PI*

Jasmin Divers^

Lorna Thorpe^

Hannah Mandel

Jennifer Truong

Shannon Wilneff Wuller

**PCORnet Core Contributors**

**Louisiana Public Health Institute:** *Tom Carton, mPI,* Anna Legrand, Elizabeth Nauman

**Weill Cornell Medicine:** *Rainu Kaushal, mPI, Mark Weiner, mPI,*

**Children’s Hospital of Philadelphia:** *L. Charles Bailey, mPI, Christopher B. Forrest, mPI,*

**Children’s Hospital of Colorado:** *Suchitra Rao, mPI*

**Data Contributors**

**Albert Einstein College of Medicine**

Selvin Soby

**Ann & Robert H. Lurie Children's Hospital of Chicago**

*Ravi Jhaveri, PI*

**Children's Hospital of Philadelphia**

*L. Charles Bailey, mPI*

*Christopher B. Forrest, mPI*

**Cincinnati Children's Hospital Medical Center**

*Nathan M. Pajor, PI*

Jyothi Priya Alekapatti Nandagopal

**Columbia University**

*Soumitra Sengupta, PI*

**Feinberg School of Medicine, Northwestern University**

*David Liebovitz, PI*

**Nationwide Children's Hospital**

*Kelly Kelleher, PI*

Yungui Huang

**Nemours/Alfred I. duPont Hospital for Children**

*H. Timothy Bunnell, PI*

**New York University Langone Health**

*Saul Blecker, PI*

Nathalia Ladino

**Ochsner Health System**

*Dan Fort, PI*

**Penn State U College of Medicine**

*Cynthia Chuang, PI*

**Seattle Children's Hospital**

*Dimitri Christakis, PI*

Daksha Ranade

**Stanford University School of Medicine**

Keith E. Morse

**Temple University**

*Shannon Herring, PI*

**The Ohio State University**

*Soledad Fernandez, PI*

Neena Thomas

**University of California San Francisco**

*Susan Kim, PI*

Mark Pletcher

**University of Colorado School of Medicine and Children’s Hospital Colorado**

*Suchitra Rao, PI*

Sara J. Deakyne Davies

**University of Michigan**

*David Williams, PI*

**University of Missouri School of Medicine**

*Xing Song, PI*

**University of Nebraska Medical Center**

*Carol Geary, PI*

Jim Svoboda

**University of Pittsburgh**

*Jonathan Arnold, PI*

*Michael Becich, PI*

*Yalini Senathirajah, PI*

Nickie Cappella

**Vanderbilt University Medical Center**

*Yacob Tedla, PI*

Wei-Qi Wei

**Weill Cornell Medicine**

*Rainu Kaushal, PI*

Thomas Campion

# Reference

1. Simon TD, Cawthon ML, Popalisky J, Mangione-Smith R, Center of Excellence on Quality of Care Measures for Children with Complex Needs (COE4CCN). Development and validation of the pediatric medical complexity algorithm (PMCA) version 2.0. *Hosp Pediatr*. 2017;7(7):373–377. doi: 10.1542/hpeds.2016-0173.

2. Rao S, Lee GM, Razzaghi H, et al. Clinical features and burden of postacute sequelae of SARS-CoV-2 infection in children and adolescents. *JAMA pediatrics*. 2022;176(10):1000–1009.

3. Psaty BM, Koepsell TD, Lin D, et al. Assessment and control for confounding by indication in observational studies. *J Am Geriatr Soc*. 1999;47(6):749–754. doi: 10.1111/j.1532-5415.1999.tb01603.x.

4. Kyriacou DN, Lewis RJ. Confounding by indication in clinical research. *JAMA*. 2016;316(17):1818–1819. doi: 10.1001/jama.2016.16435.

5. Walker AM, Patrick AR, Lauer MS, et al. A tool for assessing the feasibility of comparative effectiveness research. *Comparative effectiveness research*. 2013:11–20.

6. OHDSI. Chapter 12: Population-level estimation. The Book of OHDSI Web site. <https://ohdsi.github.io/TheBookOfOhdsi/PopulationLevelEstimation.html>. Updated 2021.

7. Schuemie MJ, Ryan PB, DuMouchel W, Suchard MA, Madigan D. Interpreting observational studies: Why empirical calibration is needed to correct p‐values. *Stat Med*. 2014;33(2):209–218.

8. Schuemie MJ, Hripcsak G, Ryan PB, Madigan D, Suchard MA. Empirical confidence interval calibration for population-level effect estimation studies in observational healthcare data. *Proceedings of the National Academy of Sciences*. 2018;115(11):2571–2577.
