# Supplementary material for: Post-Acute Dyslipidemia and Abnormal Body Mass Index in Children and Adolescents with COVID-19: A Cohort Study from the RECOVER Initiative
Source: J Pediatr. Author manuscript; Available in PMC 2026 May 30. (PMC13221949; doi:10.1016/j.jpeds.2026.114996)
Supplement: 2 [file NIHMS2168194-supplement-2.docx]

**Table S1**. **Variables definitions used in the study evaluating the relative risk of post-acute dyslipidemia and abnormal BMI outcomes after SARS-CoV-2 infection in children and adolescents.**

| Variable | Functional form | Values | Detail | Codes/references |
| --- | --- | --- | --- | --- |
| Treatment  (i.e., Exposure) |  |  |  |  |
| Documented SARS-CoV-2 infection | Indicator | Yes/No | Based on the observation and visit occurrence domains. Defined as a polymerase-chain-reaction (PCR), serology, or antigen tests positive for COVID-19, or diagnoses of COVID-19, post-acute sequelae of SARS-CoV-2 (PASC) regardless of the presence of symptoms. | See <https://github.com/PEDSnet/PASC/tree/main/observation_derivation_recover_ml_phenotype/specs> for detailed codes. |
| Outcome |  |  |  |  |
| Abnormal Total cholesterol (TC) | Indicator | Yes/No | TC ≥ 200 mg/dL | See <https://github.com/lyqlei/PASC-Dyslipid_BMI/blob/74d6b87071643ef513482ccaf70f5ed444cc29fd/codeset_measurement_table_dyslipid.csv> for detailed SNOMED concept codes. |
| Abnormal Triglycerides (TG) | Indicator | Yes/No | TG:   - ≥ 100 mg/dL, 0-9 years; - ≥ 130 mg/dL, 10-19 years; - ≥ 150 mg/dL, 20-21 years |  |
| Abnormal low-density lipoprotein (LDL) cholesterol | Indicator | Yes/No | LDL cholesterol ≥ 130 mg/dL |  |
| Abnormal high-density lipoprotein (HDL) cholesterol | Indicator | Yes/No | HDL cholesterol < 40 mg/dL |  |
| Abnormal non-HDL cholesterol | Indicator | Yes/No | Non-HDL ≥ 145 mg/dL |  |
| Any abnormal lipid laboratory result | Indicator | Yes/No | Including incident occurrence of any dyslipidemia outcome studied. |  |
| Abnormal BMI | Indicator | Yes/No | - BMI z-score ≥ 95^th^ percentile, 2-18 years; - BMI ≥ 130 mg/dL, 19-21 years | Measurement concept ID: 3038553 for BMI, 2000000043 for BMI z-score |
| Confounding variables |  |  |  |  |
| Age (years) | Linear | NA | Based on records in the person domain. | Age is defined as the integer of (date – birth date)/365.25 |
| Sex | Indicator | Male/Female | Based on records in the person domain. | NA |
| Race and ethnicity | 6 categories | Asian American/Pacific Islander [AAPI], Hispanic, Multiple, Non-Hispanic Black [NHB], Non-Hispanic White [NHW], and Other/Unknown | Based on records in the person domain. | NA |
| Obesity | 3 categories | Yes/No/Unknown | Based on records in the measurement domain.  If measured at age < 24*30.5 days, NHANES weight z score > 1.64  If measured at 24*30.5 < age <240*30.5, NHANES BMI z score > 1.64  If measured at age >= 240*30.5, BMI kg/m2 > 30 | NA |
| PMCA (Pediatric Medical Complexity Algorithm) | 3 categories | No chronic condition (PMCA = 0)  Non-complex chronic condition (PMCA = 1)  Complex chronic condition comorbidities (PMCA = 2) | Based on the condition occurrence and visit occurrence domains. | ^1,2^ |
| Diagnosis of each chronic condition cluster in 24 months to 7 days prior to the entry | Indicator | Yes/No | 205 chronic condition clusters were defined based on the condition occurrence and visit occurrence domains. | ^3,4^ |
| Number of visits to emergency department in 24 months to 7 days prior to the entry | 4 categories | 0/1/≥2 | Based on the condition occurrence and visit occurrence domains. | NA |
| Number of inpatient visits in 24 months to 7 days prior to the entry | 4 categories | 0/1/≥2 | Based on the condition occurrence and visit occurrence domains, including Inpatient Hospital Stay, Emergency Department Admit to Inpatient Hospital Stay, and Observation Stay | NA |
| Number of outpatient visits in 24 months to 7 days prior to the entry | 4 categories | 0/1/≥2 | Based on the condition occurrence and visit occurrence domains including Ambulatory/Outpatient Visit (With a Physician) and Interactive Telemedicine Service | NA |
| Number of unique medications in 24 months to 7 days prior to the entry | 4 categories | 0/1/≥2 | Based on the drug exposure domain. | NA |
| Number of negative COVID-19 tests in 24 months to 7 days prior to the entry | 4 categories | 0/1/≥2 | Based on the observation derivation recover domain | NA |
| Stratification variable |  |  |  |  |
| Obesity Status |  | Healthy (non-obese), Obesity class 1, Obesity class 2, Obesity class 3 | Healthy:   - BMI z-score ≥ –1.645 and < 1.036 (5^th^ to 85^th^ percentile), 0-19 years; - 18.5 ≤ BMI < 25, 20-21 years   Obesity class 1:   - BMI z-score > 1.645 (95^th^ percentile), 0-19 years; - 30 ≤ BMI < 35, 20-21 years   Obesity class 2:   - 120% of 95^th^ percentile for sex/age ≤ BMI < 140% of 95^th^ percentile for sex/age, 0-19 years - 35 ≤ BMI < 40, 20-21 years   Obesity class 3:   - BMI ≥ 140% of 95^th^ percentile for sex/age -OR- BMI ≥ 40, 0-19 years - BMI ≥ 40, 20-21 years | BMI z-score classification was based on age- and sex-specific CDC 2000 growth charts for participants aged 2 to 19 years |
| Other variables for eligibility criteria |  |  |  |  |
| Prior encounter in 24 months to 7 days prior to the entry | Indicator | Yes/No | Based on the condition occurrence and visit occurrence domains. | NA |
| Follow-up encounter in 28 to 179 days after the entry | Indicator | Yes/No | Based on the condition occurrence and visit occurrence domains. | NA |

*Note: All the domains in the table above are based on the PEDSnet common data model (CDM). More details are available through this link:* [*https://data-models-service.research.chop.edu*](https://data-models-service.research.chop.edu)*.*

**Reference:**1. Simon TD, Cawthon ML, Popalisky J, Mangione-Smith R, Center of Excellence on Quality of Care Measures for Children with Complex Needs (COE4CCN). Development and validation of the pediatric medical complexity algorithm (PMCA) version 2.0. *Hosp Pediatr*. 2017;7(7):373–377. doi: 10.1542/hpeds.2016-0173.

2. Rao S, Lee GM, Razzaghi H, et al. Clinical features and burden of postacute sequelae of SARS-CoV-2 infection in children and adolescents. *JAMA pediatrics*. 2022;176(10):1000–1009.

3. Psaty BM, Koepsell TD, Lin D, et al. Assessment and control for confounding by indication in observational studies. *J Am Geriatr Soc*. 1999;47(6):749–754. doi: 10.1111/j.1532-5415.1999.tb01603.x.

4. Kyriacou DN, Lewis RJ. Confounding by indication in clinical research. *JAMA*. 2016;316(17):1818–1819. doi: 10.1001/jama.2016.16435.
