# Supplementary material for: Post-Acute Dyslipidemia and Abnormal Body Mass Index in Children and Adolescents with COVID-19: A Cohort Study from the RECOVER Initiative
Source: J Pediatr. Author manuscript; Available in PMC 2026 May 30. (PMC13221949; doi:10.1016/j.jpeds.2026.114996)
Supplement: 3 [file NIHMS2168194-supplement-3.docx]

**Table S2. Calibrated adjusted relative risks of post-acute dyslipidemia and abnormal BMI outcomes in COVID-19-positive versus COVID-19-negative cohorts after negative-control outcome calibration.**

| **Outcome Name** | **aRR (95% CI)** | **P-value** |
| --- | --- | --- |
| **Dyslipidemia Outcomes** | | |
| Abnormal TG | 1.18 (0.89-1.54) | 0.247 |
| Abnormal HDL | 1.14 (0.87-1.50) | 0.336 |
| Abnormal LDL | 1.09 (0.82-1.44) | 0.547 |
| Abnormal TC | 1.05 (0.80-1.38) | 0.722 |
| Abnormal Non-HDL | 0.95 (0.71-1.27) | 0.726 |
| **Composite dyslipidemia outcomes** | | |
| Any abnormal lipid result | 1.14 (0.87-1.49) | 0.358 |
| **Abnormal BMI Outcomes** | | |
| Abnormal BMI | 1.05 (0.77-1.42) | 0.772 |

*Calibrated adjusted relative risks (aRR) and 95% confidence intervals for incident post-acute dyslipidemia and abnormal BMI outcomes. Estimates were calibrated using negative-control outcomes to account for potential residual confounding. †Definitions and abbreviations: Abnormal HDL Cholesterol: high-density lipoprotein (HDL) cholesterol: < 40 mg/dL; Abnormal LDL Cholesterol: low-density lipoprotein (LDL) cholesterol: ≥ 130 mg/dL; Abnormal Non-HDL Cholesterol: non-HDL cholesterol: ≥ 145 mg/dL; Abnormal TC: total cholesterol (TC): ≥ 200 mg/dL; Abnormal TG: triglycerides (TG) ≥ 100 mg/dL for ages 0-9 years, ≥ 130 mg/dL for ages 10-19 years, ≥ 150 mg/dL for ages 20-21 years. Any abnormal lipid lab results: the first occurrence of any of the above thresholds during the study period; Abnormal BMI: BMI z-score≥ 95th percentile for ages 2-18 years, BMI ≥ 30 kg/m2 for ages 19-21 years. aRR: adjust Relative Risk.*
