# Supplementary material for: Post-Acute Dyslipidemia and Abnormal Body Mass Index in Children and Adolescents with COVID-19: A Cohort Study from the RECOVER Initiative
Source: J Pediatr. Author manuscript; Available in PMC 2026 May 30. (PMC13221949; doi:10.1016/j.jpeds.2026.114996)
Supplement: 4 [file NIHMS2168194-supplement-4.docx]

**Table S3. Adjusted relative risk of post-acute dyslipidemia and abnormal BMI outcomes in COVID-19-positive versus COVID-19-negative cohorts stratified by baseline obesity status**

| **Stratification Analysis** | **Incidence in COVID-19 Cohort** | **Incidence in Negative Cohort** | **aRR**  **(95% CI)** | **P-value** |
| --- | --- | --- | --- | --- |
| 1. **Healthy Weight** | **N=327,501** | **N=939,834** |  | |
| **Abnormal TG** | 0.35% | 0.25% | 1.28 (1.19-1.38) | <0.001 |
| **Abnormal HDL** | 0.42% | 0.32% | 1.20 (1.12-1.29) | <0.001 |
| **Abnormal LDL** | 0.14% | 0.10% | 1.20 (1.07-1.36) | <0.01 |
| **Abnormal TC** | 0.20% | 0.16% | 1.14 (1.03-1.26) | 0.01 |
| **Abnormal Non-HDL** | 0.08% | 0.06% | 1.08 (0.93-1.27) | 0.31 |
| **Any abnormal lipid result** | 0.71% | 0.53% | 1.23 (1.17-1.30) | <0.001 |
| 1. **Obesity (Class 1-3)** | **N=384,289** | **N=1,080,413** |  | |
| **Abnormal TG** | 0.49% | 0.34% | 1.28 (1.21-1.36) | <0.001 |
| **Abnormal HDL** | 0.58% | 0.41% | 1.25 (1.18-1.32) | <0.001 |
| **Abnormal LDL** | 0.20% | 0.15% | 1.19 (1.08-1.30) | <0.001 |
| **Abnormal TC** | 0.27% | 0.21% | 1.14 (1.06-1.24) | <0.001 |
| **Abnormal Non-HDL** | 0.11% | 0.09% | 1.06 (0.93-1.20) | 0.38 |
| **Any abnormal lipid result** | 0.95% | 0.69% | 1.24 (1.18-1.29) | <0.001 |
| 1. **Class 3 Obesity** | **N=4,417** | **N=8,833** |  | |
| **Abnormal TG** | 4.24% | 3.42% | 1.35 (1.09-1.68) | <0.01 |
| **Abnormal HDL** | 5.19% | 4.77% | 1.17 (0.96-1.42) | 0.11 |
| **Abnormal LDL** | 1.39% | 1.56% | 0.87 (0.63-1.22) | 0.43 |
| **Abnormal TC** | 1.51% | 1.53% | 1.00 (0.72-1.39) | 0.99 |
| **Abnormal Non-HDL** | 0.63% | 0.90% | 0.65 (0.41-1.04) | 0.07 |
| **Any abnormal lipid result** | 7.08% | 6.57% | 1.12 (0.95-1.34) | 0.18 |

*Adjusted relative risks (aRR) and 95% confidence intervals for incident post-acute dyslipidemia and abnormal BMI outcomes, stratified by baseline obesity status. Results are shown separately for individuals who were health weight, with obesity (Class 1-3), and those with Class 3 obesity at baseline. †Definitions and abbreviations: Abnormal HDL Cholesterol: high-density lipoprotein (HDL) cholesterol: < 40 mg/dL; Abnormal LDL Cholesterol: low-density lipoprotein (LDL) cholesterol: ≥ 130 mg/dL; Abnormal Non-HDL Cholesterol: non-HDL cholesterol: ≥ 145 mg/dL; Abnormal TC: total cholesterol (TC): ≥ 200 mg/dL; Abnormal TG: triglycerides (TG) ≥ 100 mg/dL for ages 0-9 years, ≥ 130 mg/dL for ages 10-19 years, ≥ 150 mg/dL for ages 20-21 years. Any abnormal lipid lab results: the first occurrence of any of the above thresholds during the study period; Abnormal BMI: BMI z-score≥ 95th percentile for ages 2-18 years, BMI ≥ 30 kg/m2 for ages 19-21 years. aRR: adjust Relative Risk.*
