# Supplementary figures and images for: Post-Acute Dyslipidemia and Abnormal Body Mass Index in Children and Adolescents with COVID-19: A Cohort Study from the RECOVER Initiative
Source: J Pediatr. Author manuscript; Available in PMC 2026 May 30. (PMC13221949; doi:10.1016/j.jpeds.2026.114996)

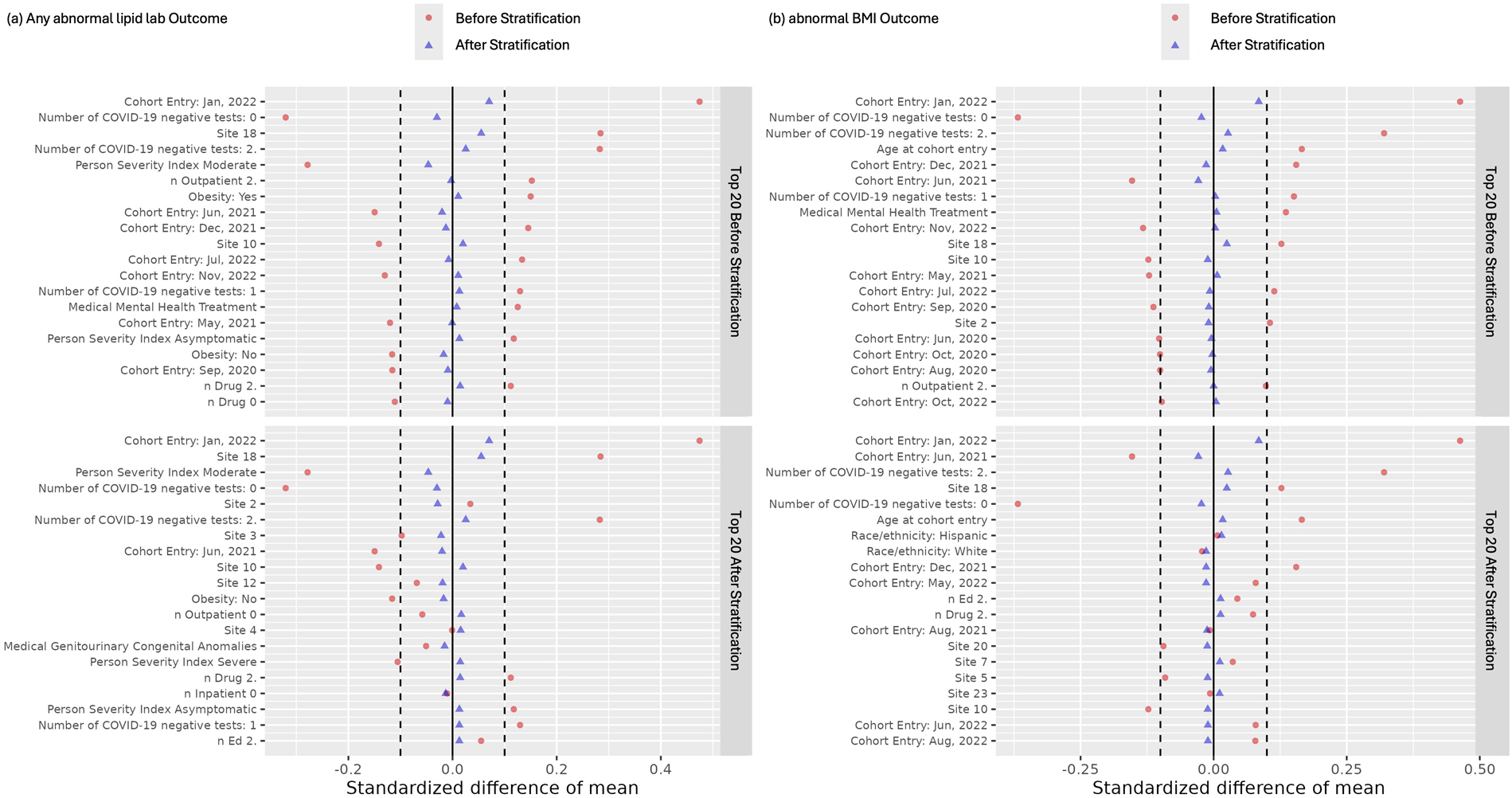

Supplement: 5 [file NIHMS2168194-supplement-5.jpg]
